# Primary care physician responses to requests by older adults for unnecessary drugs: a qualitative study

**DOI:** 10.1186/s12875-022-01857-x

**Published:** 2022-09-26

**Authors:** Zhijie Xu, Yiting Lu, Xujian Liang, Yuanqu Ye, Yang Wang, Zhiling Deng, Yuanyuan Xu, Lizheng Fang, Yi Qian

**Affiliations:** 1 Department of General Practice, The Second Affiliated Hospital, Zhejiang University School of Medicine, Hang Zhou, China; 2 Department of General Practice, Tongji University School of Medicine, Shanghai, China; 3 Department of General Practice, Sir Run Run Shaw Hospital, Zhejiang University School of Medicine, Hang Zhou, China; 4 Baili Community Healthcare Center, The People’s Hospital of Longhua, Shenzhen, China; 5 China Center for Health Development Studies, Peking University, Beijing, China; 6 The Eighth Affiliated Hospital, Sun Yat-sen University, Guangzhou, China; 7 School of Public Health, Hangzhou Normal University, NO.2318, Yuhangtang Rd, Yuhang District, Hangzhou, 311121 China

**Keywords:** Primary care physicians, Unnecessary drugs, Older adults

## Abstract

**Background:**

Unnecessary drug use can cause avoidable harm to older adults and is particularly common in primary care, but how primary care physicians (PCPs) respond to older adult requests for unnecessary drugs has not been well studied. This study is to explore PCPs’ responses to requests for unnecessary drugs from older adults, and their influencing factors and potential solutions.

**Methods:**

This qualitative study was conducted through semi-structured, in-depth interviews from January 4 to September 30, 2020 using a grounded theory methodology. A purposive sample of PCPs affiliated with community healthcare centers in Zhejiang Province and Guangdong Province, China were recruited. The face-to-face interviews were audio-recorded, transcribed verbatim, and independently coded by two investigators. Themes surrounding PCPs’ responses to requests for unnecessary drugs, their influencing factors and potential solutions were analysed using a constant comparative approach.

**Results:**

Of the 23 participants involved in this study, 12 (52%) were women and the mean age was 35 years. PCPs frequently declined older adults’ requests for unnecessary drugs through dissuasion, and occasionally rebuffed patients or referred them to another practitioner. PCPs may fulfill requests due to physician acquiescence, patient pressure, or inadequate supervision and support. Participants recommended four potential solutions to improve the quality of prescribing, including developing professional communication skills, enhancing pharmacist-physician collaboration, improving electronic prescription systems, and strengthening prescription management.

**Conclusions:**

PCPs typically deny requests by older adults for unnecessary drugs according to three main patterns, and guidance is necessary to reduce the potential for adverse consequences. Factors contributing to request fulfillment by PCPs require attention, and the potential solutions recommended by participants deserve consideration to improve the service quality of prescribing for older adults in primary care practices.

**Supplementary Information:**

The online version contains supplementary material available at 10.1186/s12875-022-01857-x.

## Introduction

Older adults consume the largest amount of drugs, many of which are unnecessary [1–4]. A drug is considered unnecessary when used in excessive dosage, for excessive duration, without adequate monitoring, without adequate indications, in the presence of adverse consequences indicating the dose should be reduced or discontinued, or any combination of the above [5]. A previous study found that 28% of older adults used at least one unnecessary drug in outpatient settings [6], which can lead to adverse clinical and socioeconomic consequences [7].

Approximately two-thirds of primary care visits involve one or more patient requests for diagnostic testing or treatment [8]. Notably, patients frequently express preferences for drugs, even when their consulting PCPs inform them the drugs are unnecessary [9]. Some older adults request drugs to share with their family members [10], while others may be affected by pharmaceutical advertisements or personal recommendations [11, 12]. Patient requests significantly impact prescribing decisions and increase the possibility of prescribing unnecessary drugs [13, 14].

Accommodating behavior by PCPs is understandable. Unfulfilled requests may decrease patient satisfaction [11, 15], deteriorate patient–physician relationships [16, 17], and have a strong emotional impact on PCPs [17]. However, a previous study found that patient satisfaction was influenced by the approach used by physicians to deny requests for antidepressants [18]. Although the factors affecting PCP request fulfillment for antidepressants and antibiotics have been explored separately [19, 20], it is necessary to expand the scope of the research to include unnecessary medication use in a larger population, and potential solutions are worth exploring.

We therefore undertook a qualitative study to understand how PCPs respond to requests by older adults for unnecessary drugs, provide insights into why PCPs fulfill these requests, and suggest potential solutions. Our goal was to inform future individual, organizational, and system-level interventions and policies to assist PCPs in addressing such unbeneficial requests.

## Methods

Our study employed a grounded theory methodology aimed at constructing a substantive theory that can have an applied impact on PCP prescribing behavior. From January 4 through September 30, 2020, two male investigators (Z.X. and Y.Y) and one female investigator (Y.L.)—all of whom served as general practitioners and have expertise in qualitative research methods—independently conducted semi-structured in-depth interviews. Prior to the interviews, all participants provided written informed consent. The reporting of our study conforms to the Consolidated Criteria for Reporting Qualitative Research (COREQ) [21]. Our study was approved by the Sir Run Run Shaw Hospital Ethics Committee and adhered to the Declaration of Helsinki [22].

### Setting and study population

We used a purposive sampling strategy to guide the selection process and invited participants personally or via telephone [21]. PCPs were selected from 15 public community healthcare centers in Zhejiang Province and Guangdong Province for maximum variation in age, education background, duration of practice, and institutions. PCPs were eligible if they had at least 2 years of clinical experience in family medicine and were providing care to patients aged 65 and older. The recruitment was not pre-planned, but proceeded iteratively alongside data analysis, and the sample size was determined based on theoretical saturation. Two physicians declined our invitations due to time constraints. Participants knew their interviewers before the study through WeChat, a mobile application of social media that has gained considerable popularity in China. Only one physician previously worked with one of the interviewers (Y.Y.). All PCPs voluntarily participated in the study and were not offered any compensation. Repeat interviews were not conducted.

### Interview guide

The interview guide was developed through pilot interviews with two PCPs and discussions among the three interviewers. The probing questions were refined iteratively in tandem with our analysis. At the beginning of each interview, the investigators presented a hypothetical scenario in which an older adult requested unnecessary drugs in the clinic to elicit participants’ views on this problem. Interview questions broadly focused on (1) how PCPs responded to requests by older adults for unnecessary drugs, (2) factors perceived by PCPs as contributors to request fulfillment, and (3) their recommendations to improve the service quality of prescribing for older adults in primary care (Additional file 1). Participants were encouraged to add comments relevant to their interviews after all prepared questions were answered.

### Data collection and analysis

We conducted face-to-face interviews in the private office space of each PCP. Individual information was collected from each participant prior to the interview. During the interview, investigators made field notes on the key information when necessary. All interviews were audio-recorded and transcribed verbatim by two investigators (Z.X. and X.L.) and then imported into MAXQDA 2020 (VERBI Software, Berlin) for analysis. Two investigators (Z.X. and Y. L.) independently reviewed the transcripts and analysed the data following a three-step coding process (open coding, axial coding, and selective coding) [23]. Through a process of line-by-line coding, the investigators identified emergent concepts and categories, and wrote memos to capture preliminary thoughts around their definitions. A preliminary codebook was generated and refined extensively during data collection and analysis. We used the constant comparative method to compare and expand existing categories and identify novel categories until no further subcategories emerged [24, 25].

Theoretical saturation occurred after 20 interviews, after which we interviewed three additional PCPs. Any discordance in data analysis was discussed and adjudicated by a third investigator (Y.Y.). After the findings were integrated and validated by all authors, we formulated a conceptual model to represent the relationships among the categories. Data were analysed from January to November of 2020. Transcripts were not returned to participants, yet four participants were sent the analysis results (i.e., main findings of the draft manuscript, and a table containing themes/subthemes, interpretations, and representative quotations) to indicate their agreement and make comments.

## Results

**Fig. 1:**
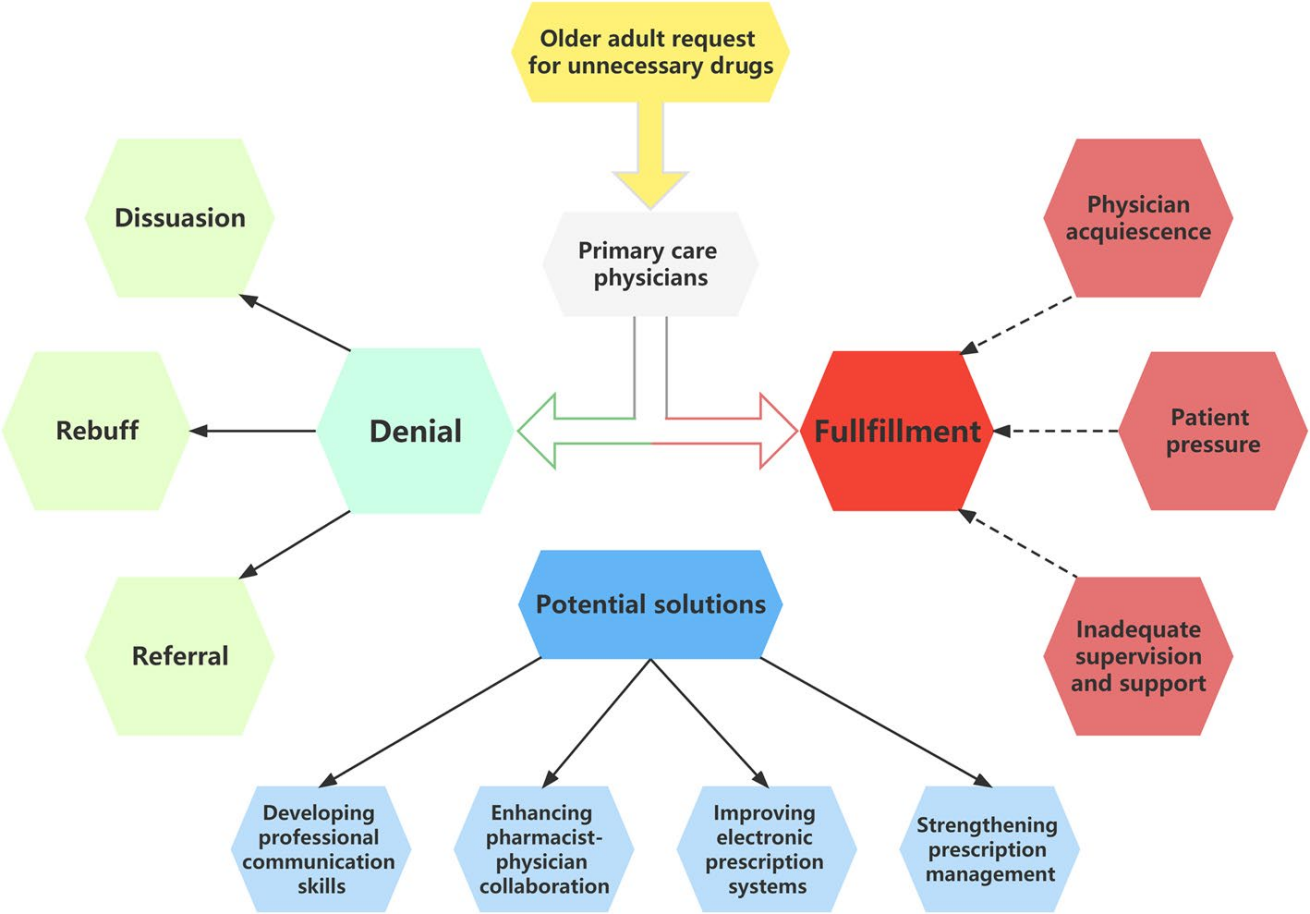
Conceptual model of PCP responses to requests for unnecessary drugs

Twenty-three PCPs were involved in our interviews, which lasted a mean of 38.3 minutes (range: 32–48 minutes). Of the 23 participants, all were Chinese Han ethnicity, 12 (52%) were women, and 7 (30%) were administrators in leadership roles. The mean age of participants was 35 years (range: 28–43 years), and their mean duration of practice was 10 years (range: 3–20 years). Participant information is presented in Additional file 2. The conceptual model is shown in Fig. 1.

### Patterns of denying requests for unnecessary drugs

#### Dissuasion

All participants agreed that they usually declined requests from older adults for unnecessary drugs and described three different patterns of denial. Dissuasion was the most common denial pattern. PCPs usually informed the patients that no indication for the drugs they requested existed, and they warned of the potential risks of drug-related harm and additional expenditure:

*‘If he had no indication to use [the antibiotics], I would make it clear that he didn’t need it when he just had a common cold. I said, the antibiotic resistance is really terrible and I didn’t expect you to have no cure someday.’ [Participant 13, woman, 37 years old]*

PCPs preferred to give striking examples of adverse drug events experienced by other patients instead of describing risk statistics. A few participants admitted that they sometimes exaggerated the side effects of drugs in order to raise patient concerns:

*‘[The old man] had a sore throat and requested an intravenous drip. I told him the fluid would enter the bloodstream and accelerate ageing of his blood vessels that might block the vessels or cause vasculitis. Then he was scared and gave up.’ [Participant 12, man, 32 years old]*

If PCPs felt it difficult to communicate with the older adults, they may have asked family members or caregivers for assistance and dissuaded them jointly. PCPs usually contact patients’ family members and caregivers through telephone and instant message. Most participants believed that the family members and caregivers could provide important information to PCPs regarding how older adults use their drugs and were helpful in their medication therapy management. One physician recounted:

*‘Some older patients, you know, do not trust us, but they followed their caregivers or family members. So I like to communicate with their sons and daughters, sometimes via telephone, to make the patients understand my thoughts.’[Participant 17, woman, 37 years old]*

#### Rebuff

Another common pattern used by PCPs to decline requests was to rebuff. Most participants described their experience of rejecting requests without much further explanation. PCPs informed the older adults that prescribing unnecessary drugs violated healthcare policies. One participant said he felt displeased when he was requested to violate the regulations to prescribe an unnecessary drug:

*‘Generally speaking, I rebuffed their requests for unnecessary drugs. If I prescribed those drugs, I had to fabricate a diagnosis. I hate breaking the regulation, so I rebuffed them.’[Participant 16, man, 31 years old]*

Some participants reported a restriction on prescribing drugs in excessive dosage or for excessive duration in their practices. They told the patients that the pharmacists will not dispense unnecessary drugs and simply said no if older adults request.

Moreover, the participants expressed their concerns about being fined if they prescribed unnecessary drugs, which were frequently shared with the older adults when declining unreasonable requests. PCPs also tried to maintain a stable relationship with the older adults, and vice versa. Many older adults were willing to understand PCPs’ concerns of penalties and withdrew their requests, although they did not realise the actual problems of using unnecessary drugs:

*‘If you want me to prescribe [the antibiotics], you need a blood test first. It’s our rules. The pharmacists will not dispense the antibiotics without a laboratory test even if I prescribed on the electronic system.’ [Participant 11, woman, 34 years old]*

*‘One reason that I often succeed in declining patient requests is that I informed them our penalty system. Most older adults can understand the direct result that I would be fined and they are unwilling to damage our relationship.’[Participant 5, man, 32 years old]*

#### Referral

The third pattern of denial was classified as a referral. Considering the difficulties in communication and concerns regarding decreasing patient satisfaction, PCPs tended to turn down older adult requests indirectly. For example, PCPs usually referred the older adults to specialists and pharmacists for their wanted medications:

*‘Sometimes I referred my patients to the hospital for further examination when they requested some drugs that had been used for a long time. Sometimes I told them that they might take a chance at the drugstores.’[Participant 10, woman, 29 years old]*

However, participants agreed that this suggestion might lead to low-value care because most drugstores in China were driven by commercial interests and incapable of providing high-quality pharmacy services. One physician acknowledged that she made excuses to avoid fulfilling the requests in extreme circumstances. Participants thought that it was a reflection of the dilemma in maintaining the balance between following the ethical principle of “Do not harm” and satisfying older adults when they faced requests for unnecessary drugs:

*‘I would find some excuses [to decline the requests], like ‘it’s out of stock’, and I suggested them to the community pharmacies for consultation. Honestly, It’s not very appropriate, but I have few choices in the crowded clinic.’[Participant 3, man, 36 years old]*

### Factors contributing to request fulfillment for unnecessary drugs

#### Physician acquiescence

All participants had at some point granted older adults’ requests for unnecessary drugs, but the contributing factors varied. Many participants argued that they only prescribed drugs commonly used in clinical practice or with few side effects. A few physicians emphasised their caution regarding the dosage and duration of unnecessary antibiotic use. One participant mentioned that although some drugs lacked evidence of symptom relief, they may provide psychological comfort. PCPs usually declined requests for unnecessary antibiotics and opioids, whereas they tended to grant requests for other drugs:

*‘I would grant their requests if I thought firstly, the drug causes no harm, and secondly, if the drug is not used on himself. It may be used to help other family members, for example, it is ok to help someone with the over-the-counter drugs, like vitamins.’ [Participant 21, woman, 36 years old]*

Most participants stated that concerns over deteriorated relationships with the older adults were the dominant reason for fulfilling requests for unnecessary drugs. They worried that a deteriorated relationship may lower patient adherence to disease management and damage the physicians’ reputations:

*‘Chronic diseases management for older adult is one of the most important tasks for we family physicians. I need to follow up and record their conditions. So if I declined the requests and annoyed an old man, maybe he would not choose me to manage his disease, then my task cannot be accomplished, right?’[Participant 2, woman, 42 years old]*

Furthermore, it requires additional time and energy to communicate with the older adults, and the process can be complex:

*‘It’s difficult [to communicate with the older adults]. They have difficulties in understanding what you mean, and some have hearing loss. It’s not one or two sentences that could convince them, but the time is very limited.’ [Participant 15, man, 38 years old]*

#### Patient pressure

Participants reported that some older adults demanded unnecessary drugs and ignored attempts at dissuasion and explanation by PCPs. Many older adults had limited understanding of their requested drugs and healthcare policies and were reluctant to learn relevant information. Some participants experienced pressure from older adults with aggressive behaviors:

*‘The old woman became abusive when I repeatedly rejected her requests. Then she cried loudly, and threw things at me. I had no way but compromise.’[Participant 1, woman, 36 years old]’*

A few participants mentioned that they might receive patient complaints and have to spend more time explaining to the administrators and patients:

*‘Then they might complain to the administrators in our practice or called the complaints hotline..I have so many patients to serve, how could I have extra time to deal with such complainants?’[Participant 4, man, 37 years old]*

Additionally, the pressure may come from the unstable health status of older adults. One physician was very worried about the consequences of older adults losing their temper:

*‘I knew that her heart was not well. If I stuck it out and displeased her, what if she had a heart attack?’[Participant 11, woman, 34 years old]*

#### Inadequate supervision and support

Participants noted several factors related to their practices that contributed to request fulfillment. In some areas, the practices lacked effective supervision measures to control the service quality of prescribing drugs for older adults. Two main causes were mentioned by participants. First, the importance of appropriate medication use for older adults was overlooked by some administrators, and second, many administrators had few tools to measure the service quality of prescribing and find the gaps:

*‘Our prescriptions are reviewed by the pharmacists monthly in my practice. But the problem is, only a very small proportion of prescriptions are reviewed that can hardly expose the errors. Very few physicians received warnings or other kinds of punishment.’[Participant 3, man, 36 years old]*

Some practices organised pharmacists to review prescriptions regularly, though without much success. The low price of breaking the rules of prescribing behavior may fail to attract the attention of PCPs. Another barrier many participants mentioned was that they lacked professional support to address requests from older adults for unnecessary drugs. As one participant expressed her wish:

*‘The [number of] pharmacy staffs in our practice is very limited. They are all very busy and incapable to counsel older patients. We are short of qualified clinical pharmacists who can help us mange the medication use for them.’[Participant 6, woman, 32 years old]*

### Potential solutions

Participants made several recommendations to assist PCPs in coping with requests from older adults for unnecessary drugs. Participants unanimously agreed that developing professional communication skills should be prioritised for PCPs. Some participants recommended that PCPs should question older adults about their need for a drug and then use communication strategies accordingly:

*‘Before we try to dissuade the patients, it’s important to listen, and understand why they want these drugs, and consider what we can do for them at present. So they will not feel that they are ignored.’[Participant 15, man, 38 years old]*

Several emphasised the importance of enhancing collaboration between pharmacists and PCPs, and voiced a wish that primary care practices would recruit and train more qualified clinical pharmacists to provide pharmaceutical care and help reduce the prescribing of unnecessary drugs by PCPs:

*‘The collaboration between pharmacists and physicians in the primary care practices, compared to other health professionals, I mean, are not very satisfactory. Pharmacists are indispensable if you want to solve this problem [of requests for unnecessary drugs]...we have difficulties in coping with the unreasonable requests without their help.’[Participant 8, woman, 34 years old]*

*‘To attract the pharmacists to primary care practices, firstly we need to increase their remuneration. And another thing is, providing them the opportunities and platform to do something technical, not just dispensing drugs.’[Participant 20, woman, 38 years old]*

Many participants expressed a desire for an improved electronic prescription system that could alert PCPs to inappropriate medication use in a timely manner and restrict them from prescribing unnecessary drugs. There is also a need for shared data among the prescription systems of medical institutions to help physicians identify drugs inappropriately used by patients:

*‘The patient may receive the drugs a few days a ago from another hospital and request you to prescribe them. I think data should be shared among the prescription systems so physicians could identify these drugs have been overused.’[Participant 9, man, 31 years old]*

Finally, most participants had similar beliefs about strengthening PCP prescription management. As recommended by one participant, the incentive mechanism should motivate PCPs to focus on providing good quality services of prescribing:

*‘The drug mark-ups as a source of financing, you know, has been stopped for years...The incentive system should not encourage physicians to prescribe more drugs. It should be linked to the service quality of prescribing instead.’[Participant 5, man, 32 years old]*

## Discussion

In this qualitative study, we characterised three patterns used by PCPs to deny older adults’ requests for unnecessary drugs and three overall factors that contribute to fulfillment of these requests. In most cases, PCPs declined requests from older adults for unnecessary drugs through dissuasion and explanation, and in some cases, they rebuffed patients or referred them to another practitioner. Meanwhile, PCPs may fulfill requests based on multiple contributing factors, including physician acquiescence, patient pressure, and inadequate support and supervision. In addition, we identified four potential solutions to improve the service quality of prescribing for older adults in primary care.

Our findings suggest that the patterns of denial depend on patient characteristics and the working environment of the PCPs. We found that PCPs tended to explain the adverse consequences of unnecessary drugs to dissuade patients who were easier to communicate with and decline the requests directly or indirectly when they were busy. Alarmingly, referrals seem to save PCPs’ time without dissatisfying patients; however, this may lead to patients receiving low-value care, including unnecessary medical examinations and therapies in hospitals and even drugstores. The adverse effect of such response can be easily ignored, and thus needs further exploration.

Most participants affirmed the importance of improving their communication skills. In Lewin’s survey study, patient satisfaction increased when physicians explained why drugs were not appropriate for them, compared to outright denial [11]. Breivold et al. found that PCPs’ perceived professional communication skill were a facilitator in handling unreasonable patient requests [26]. The positive effects of multicomponent PCP training on older adults were also observed in several intervention studies [27, 28]. Our study found that many PCPs tried to understand patient motivation before responding to requests. These findings emphasise the need to develop communication strategies to effectively deny unbeneficial requests from older adults.

This study confirmed that physician acquiescence was an important factor that contributed to request fulfillment. We found that PCPs fulfilled requests for unnecessary drugs to maintain a friendly relationship with the older adults or avoid time-consuming explanations, which was consistent with existing evidence [15–17, 29]. Interestingly, PCPs fulfilled patient requests selectively. For example, the requests for vitamins and OTC drugs were more likely to be fulfilled by PCPs than those for antibiotics and opioids. However, no participant mentioned that their acquiescence was influenced by pharmaceutical companies, which was contrary to the findings of the study by Campbell et al. [30]. Some PCPs controlled the total amount of unnecessary drugs to reduce the potential health risks. Our findings suggested that PCPs lack a full understanding of unnecessary drugs and ignore their non-health related impacts, such as wasting medical resources. Education is an important priority to help PCPs prescribe wisely [31]. A systematic review implied that the prescribing behaviors of PCPs could be successfully changed through academic detailing [32]. Nevertheless, evidence regarding the effect of education outreach specifically on PCPs is still limited [33].

In addition to education, strengthening prescription management in primary care merits attention. Two strategies could be considered to reduce unnecessary prescribing by PCPs. One is to prevent request fulfillment by PCPs for unnecessary drugs in advance. An electronic prescription alerting system could provide PCPs with an instant message alerting them of unnecessary drugs, and the pharmacists would be responsible for checking the appropriateness of each prescription. The other strategy is the supervision and punishment of unnecessary prescribing by PCPs. Participants in an interview study by O’Doherty et al. argued that some physicians would not adhere to regulations unless they were penalised [34]. In our study, participants mentioned the problem of insufficient penalty and supervision, and recommended that punishments could be implemented more comprehensively (e.g., recording of a demerit) to alert PCPs.

Our findings have important implications for enhancing pharmacist-physician collaboration in primary care settings to reduce unnecessary drug use. We found that many PCPs had the desire to involve pharmacists in the management of prescription drug use among the older adults. Pharmacists play an important role in assessing prescription and pharmaceutical care, such as medication counselling and medicine reviews [35–37]. They also have opportunities to work closely with PCPs to address medication management challenges [38, 39]. For example, an older adult could be referred to the pharmacist for consultation if a PCP had difficulties with an explanation. This could be achieved in pharmacist-managed clinics, but barriers to and facilitators of the implementation of pharmacy services in primary care clinics should be explored [40]. Moreover, pharmacists could provide training courses for PCPs on appropriate prescribing [41]. Further research is needed to explore the feasibility and effectiveness of cooperation between pharmacists and PCPs for handling older adults’ requests for unnecessary drugs.

### Strengths and limitations

A particular strength of our study is that three interviewers collected participants’ views collaboratively using a grounded theory methodology, which reduces the subjective bias in the analysis and interpretation of the findings. Another strength is that most PCPs have actual experiences of being requested for unnecessary drugs by older adults. The participants are willing to share their perspectives that enabled us to gather a range of views across diverse settings. Our study has several limitations. First, the study included PCPs from 15 practices in two provinces, yet our findings may not reflect the experiences of PCPs in other geographic regions. We also did not quantify how often these experiences occurred. Quantitative studies involving different practices across the country are needed to fully understand the responses and perceptions of PCPs. Second, reporting bias may exist because we only interviewed physicians. The views of other health professionals, such as pharmacists, could be elicited in future studies. Finally, the participants may have different understandings of unnecessary drugs despite the explanation prior to the interviews, which could have an impact on their responses.

## Conclusions

We found that PCPs denied requests from older adults for unnecessary drugs through dissuasion, rebuff, or referral, and that factors involving physicians, patients, and institutions could contribute to request fulfillment. The potential solutions recommended by participants deserve consideration by physicians, healthcare managers, and policymakers. A concerted effort from stakeholders will contribute to the development of interventions and policies to improve the service quality of primary care with respect to prescribing for older adults.

## Supplementary Information

**Additional file 1.** Interview guide.

**Additional file 2.** Participant information.

## Data Availability

The transcripts used during the current study are available from the corresponding author upon reasonable request.

## Abbreviations

PCPs: Primary care practitioners
OTC: Over-the-counter
