# Supplementary material for: Primary care physician responses to requests by older adults for unnecessary drugs: a qualitative study
Source: BMC Prim Care. 2022 Sep 26;23:247. doi: 10.1186/s12875-022-01857-x (PMC9511742; doi:10.1186/s12875-022-01857-x)
Supplement: Supplementary file 1 — Additional file 1. Interview guide. [file 12875_2022_1857_MOESM1_ESM.docx]

**Additional file 1** Interview Guide

**Participant ID:______**

**Introduction**

Thank you for participating in our interview! I'd like to know how you respond to requests by older adults for unnecessary drugs and what you think of this problem. The unnecessary drug means that any drug is used in excessive dosage; for excessive duration; without adequate monitoring; without adequate indications for its use; in the presence of adverse consequences which indicate the dose should be reduced or discontinued. There are no right or wrong answers, so please don't feel that you have to answer in a certain way. The questions are also not aimed at you, which means we don't know what the right views and decisions are. This interview is voluntary and you have the right to withdraw at any time. Also, all questions are hypothetical.

Do you have any other questions before our interview begins?

I'm going to start recording now, and I'll write down the keywords you mention.

**Scenario description**

I'd like to start this interview with a clinical scenario. It’s 10 o 'clock in the morning. You are working in the clinic as usual. Here comes a 68-year-old male patient with multiple chronic illnesses who comes to you every month for refilling drugs. His condition is well controlled, and you know his condition well. After you refill the drugs he needs for his chronic diseases, he tells you that he wants some antibiotics, such as levofloxacin, so he can take them conveniently if he has a common cold. He also wants the 'hormone cream' for his skin because he sometimes feels itchy. However, based on what you know about him, you think the drugs he requested are unnecessary.

Think about this scenario, and I'll ask you some questions next.

**General experience and attitudes**

1. Have you encountered a similar situation in your clinical practice?
2. What types of drugs that are possibly unnecessary do older adults often request you to prescribe?
3. What do you think of older adults requesting you to prescribe unnecessary drugs?

-Do you feel concerned about the consequences of older adult requests for unnecessary drugs?

-If yes, can you talk about your concerns?

1. Do you think it is difficult to communicate with older adults about drug use? Why?

**Responses to older adult requests**

1. What did you usually do when the older adults requested you to prescribe unnecessary drugs?
2. Do you think you can decline older adult requests for unnecessary drugs in most cases?

-If not, what is the potential reasons?

1. What barriers do you encounter when you tried to decline patient requests for unnecessary drugs?
2. Under what circumstances did you decline patient requests for unnecessary drugs?

-Can you talk about the reasons behind this?

1. Can you share your successful experience of declining the requests?
2. Under what circumstances did you fulfill patient requests for unnecessary drugs?

-Can you talk about the reasons behind this?

1. Have your fulfillment led to some adverse consequences?

-If yes, can you describe the consequences?

**Recommendations for solutions**

1. What kinds of measures do you think may help solve this problem?

-What do you think can primary care physicians do? Why?

-What do you think can clinical leaders of the primary care practices do? Why?

-What do you think can policy-makers do? Why?

**Ending words**

I have finished all my questions. Do you have any other comments on this interview?

Thank you for your participation! After all the interviews and data analysis were completed, I may invite you to review and discuss the results of our results. I hope we can keep in touch.
