# Supplementary material for: Primary care physician responses to requests by older adults for unnecessary drugs: a qualitative study
Source: BMC Prim Care. 2022 Sep 26;23:247. doi: 10.1186/s12875-022-01857-x (PMC9511742; doi:10.1186/s12875-022-01857-x)
Supplement: Supplementary file 2 — Additional file 2. Participant information. [file 12875_2022_1857_MOESM2_ESM.docx]

**Additional file 2** Participant information

| **ID** | **Sex** | **Age, y** | **Area** | **Degree** | **Practice in the city** | **Years in**  **Practice** | **Administrative**  **tasks** |
| --- | --- | --- | --- | --- | --- | --- | --- |
| 001 | F | 36 | G | Bachelor | Yes | 11 | No |
| 002 | F | 43 | G | Master | Yes | 18 | Yes |
| 003 | M | 36 | G | Bachelor | Yes | 12 | No |
| 004 | M | 37 | G | Master | No | 11 | Yes |
| 005 | M | 32 | Z | Master | No | 4 | No |
| 006 | F | 32 | Z | Master | Yes | 5 | No |
| 007 | M | 36 | Z | Bachelor | Yes | 10 | No |
| 008 | F | 34 | G | Bachelor | Yes | 7 | No |
| 009 | M | 31 | Z | Master | Yes | 5 | Yes |
| 010 | F | 29 | Z | Bachelor | Yes | 4 | No |
| 011 | F | 34 | Z | Bachelor | Yes | 10 | No |
| 012 | M | 32 | Z | Bachelor | Yes | 9 | No |
| 013 | F | 37 | Z | Master | Yes | 11 | Yes |
| 014 | M | 33 | Z | Bachelor | Yes | 9 | No |
| 015 | M | 38 | Z | Bachelor | Yes | 15 | No |
| 016 | M | 31 | G | Master | Yes | 12 | Yes |
| 017 | F | 37 | G | Bachelor | Yes | 5 | No |
| 018 | M | 34 | G | Bachelor | Yes | 12 | No |
| 019 | F | 32 | G | College | Yes | 15 | No |
| 020 | F | 41 | G | Master | Yes | 9 | Yes |
| 021 | F | 36 | G | Master | Yes | 7 | Yes |
| 022 | F | 29 | G | Bachelor | Yes | 3 | No |
| 023 | M | 35 | G | Bachelor | Yes | 20 | No |
| F = female; M = male; G=Guangdong; Z=Zhejiang | | | | | | | |
